# Evaluation of nutritional and functional properties of anatomical parts of two sorghum (*Sorghum bicolor*) varieties

**DOI:** 10.1016/j.heliyon.2023.e17296

**Published:** 2023-06-14

**Authors:** Janet Adeyinka Adebo, Hema Kesa

**Affiliations:** Food Evolution Research Laboratory, School of Hospitality and Tourism, College of Business and Economics, University of Johannesburg, South Africa

**Keywords:** Cereal, Anatomical parts, Food analysis, Cultivars

## Abstract

Compositional differences exist among grain varieties as well as in the content of respective fractions. The proximate composition, amino acids, mineral contents, and functional properties of white and brown sorghum and its anatomical parts (dehulled and bran) were studied. The results showed that the bran had higher crude protein, crude fat, crude fibre, and ash contents for both sorghum varieties than the whole grain and dehulled samples. Likewise, significantly (p ≤ 0.05) higher essential and non-essential amino acids and minerals, particularly calcium, zinc, potassium were recorded for the bran samples compared to the whole grains or dehulled ones. With regard to the functional properties, the hydration capacity, hydration index, water, and oil absorption capacities of the dehulled samples were significantly (p ≤ 0.05) lower than the other investigated samples, except for bulk density, which was significantly (p ≤ 0.05) higher. In contrast, none of the swelling capacities differed significantly in any of the samples. In conclusion, sorghum bran has significant potential in the food industry and could be an excellent material for formulating high-fibre foods and serving as a nutritionally-rich food ingredient.

## 1 Introduction

The vital role of food in the daily diet cannot be over-emphasized, as food is needed for satisfying hunger and providing nutrients required for the effective functioning of the human body. Cereals (of the Grains food group) belong to the grass family called *Gramineae* [1]. According to Olugbire et al. [2], cereals are a major food staple globally, significantly contributing to a large percentage of daily food requirements. As noted by Awika [3], about 60% of required calories in developing countries are derived from cereals. While notable cereals such as wheat, maize, and rice contribute more than half of calories consumed globally, minor cereals such as millet and sorghum are also key contributors to calorific intake in developing countries [4].

Sorghum (*Sorghum bicolor*) is the fifth most important food crop after rice, maize, wheat, and barley, and an important food source for millions of people in Africa and Asia. Growing sorghum over other grains has several benefits, such as improved yields in dry and arid regions [5,6]. In African countries, for example, South Africa, sorghum is usually grown in drought-prone areas, is regarded as the second most significant cereal crop, and serves as a staple food in rural communities, representing their primary source of nutrients and energy [7].

The end use of the sorghum grain largely depends on the grain type and quality. Sorghum grains are spherical, and come in different sizes and colours ranging from dark brown, red, white, tan and pale orange to brownish red. Brown, white, and red are the available grains sold commercially [8]. The sorghum caryopsis consists of three distinctive anatomical components, namely, the pericarp, germ, and endosperm, constituting 6.5, 9.4 and 84.2% of the total grain, respectively [9,10]. The relative proportion of these sorghum caryopses varies depending on variety and environment [9], while the pericarp colour is controlled genetically by the R and Y genes [11].

The preparation of sorghum into foods such as porridge or beverages generally begins with milling, before the resulting flour is processed. Milling separates the outer fibrous pericarp and oil-rich germ from the starchy endosperm [12]. Traditionally, the sorghum grain was milled by hand in rural households, by pounding the grains in a wooden pestle and mortar; this process, however, is fast declining and is becoming replaced by more mechanized milling systems (hammer mill and abrasive dehuller or decorticator) [12,13]. During the milling of sorghum, the pericarp (bran) is often discarded as waste, despite its beneficial constituents. According to Taylor and Kruger [13], bran removal or reduction in bran particle size due to milling affects the nutritional quality of sorghum, and is reported to affect the bioavailability of some nutrients such as amino acids, starch, dietary fibres and so on. This reduction of nutrients in sorghum brans has become a matter of nutritional concern, as there is strong scientific evidence linking regular consumption of whole grain cereal foods to long-term health benefits [14,15].

With a continually growing population in Africa [16] there will be a need for reliance on indigenous grains such as sorghum. In addition, there is growing interest in sorghum as a food ingredient in Western societies which currently do not generally consume this grain [17]. The food industry is also constantly seeking cheaper, readily accessible, gluten-free, and viable alternative grains which can be developed and utilized for food use. This study thus investigated the nutritional quality and functional properties of two sorghum varieties.

## 2 Materials and methods

### 2.1 Materials and sample preparation

**Fig. 1:**
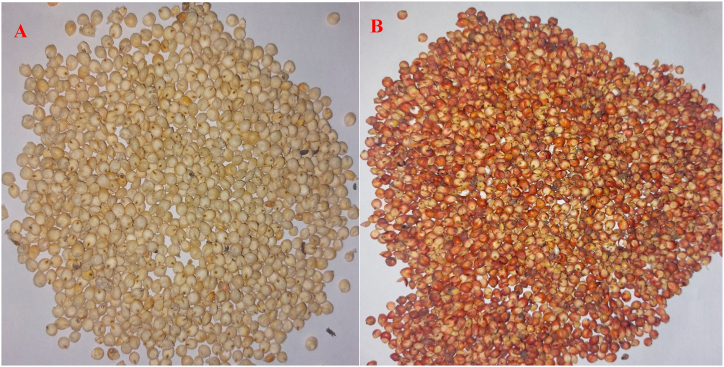
(A) White and (B) brown sorghum types. (For interpretation of the references to colour in this figure legend, the reader is referred to the Web version of this article.)

White and brown sorghum (*Sorghum bicolor*) (Fig. 1) types were purchased separately from a local market in Johannesburg, South Africa. The grains were cleaned and screened to remove extraneous material and debris. A portion of both sorghum grains (white and brown) was dehulled with a Prairie Research Laboratory (PRL) type abrasive dehuller (Rural Industries Innovation Centre, Kanye, Botswana). Briefly, 10 kg of each type of cleaned sorghum grain were fed into the dehuller barrel through a hopper fitted with a flow regulator. Subsequent to the dehulling process, the bran was removed utilizing a cyclone fan and collected. The decorticating time ranged from 3 to 8 min, depending on the sorghum type and the operator’s satisfaction. The sorghum grains and the respective parts (bran and dehulled grain) were collected separately and packed into ziplock bags. Milling was done using a laboratory mill (Perten Laboratory Mill 3600, Perten Instruments, Stockholm, Sweden) and the milled samples were passed through a 500 μm sieve. The sorghum grains were collected to obtain the white whole grain sorghum flour (WSF), white dehulled sorghum flour (WDF), brown whole grain sorghum flour (BSF) and brown dehulled flour (BDF). The white sorghum bran (WSB) and brown sorghum bran (BSB) were already in a powdered (flour) form. All samples were subjected to further analysis as described in succeeding sections.

### 2.2 Proximate compositions and total energy of the sorghum flours

The proximate compositions (moisture crude protein, crude fat, crude fibre, and ash content) were determined respectively using methods 934.01, 990.03, 920.39 (A), 978.10 and 923.03 (32.1.05) of AOAC (2006). Total carbohydrate was determined by difference (percentages of moisture, fat, ash, fibre and protein subtracted from 100%) [18], while total energy was calculated using the Atwater factors as stated below [19]

(1) Energy value (kcal) = % Protein × 4 + % Carbohydrate × 4 + % Fat × 9.0.

### 2.3 Amino acid composition

Amino acid composition of all the flour samples (WSF, WDF, WSB, BSF, BDF and BSB) was determined at the Agricultural Research Council (ARC)-Irene Analytical Research Laboratory, Pretoria, South Africa, using High-Performance Liquid Chromatography (HPLC) following the procedure described by Adebiyi et al. [20]. The sorghum samples were hydrolysed using an equal volume of 6 N HCl solution and an internal standard (a-amino-b-guanidino propionic acid) for 24 h at 1150 °C, and allowed to cool. The hydrolysate was then transferred to an Eppendorf tube and centrifuged at 3000 rpm for 10 min, after which the supernatant (protein hydrolysate) was filtered (0.45 μm). The protein hydrolysate was later dried under nitrogen gas and derivatized using FMOC reagent (9-flourenylmethyl chloroformate) and borate buffer. After a few seconds, the mixture was extracted with pentane. The derivatized extract was then analyzed on HPLC using fluorescence detectors (Schoeffel FS 970, Perkin-Elmer LS-4 and Shimadzu RF-530 at excitation and emission wavelengths of 260 and 313 nm). A mixture of HPLC grade acetonitrile, methanol and acetic acid (10:40:50, v/v/v) was used as the eluent, which was varied linearly to acetonitrile:acetic acid (50:50, v/v) over 90 min. Oven temperature was kept at 40 °C, while the gradient flow was initiated at 3 min, at a 1.3 mL/min flow rate. The flow rate was later increased to 2 mL/min for 0.5 min at the end of the gradient. Using calibration curves from amino acid standards, (Sigma Aldrich, St. Loius, USA), the concentrations of the amino acids in the samples were determined.

### 2.4 Determination of mineral element composition using inductively coupled plasma optical emission spectrometry (ICP-OES) and inductively coupled plasma mass spectrometry (ICP-MS)

#### 2.4.1 Microwave digestion

Using the method described in Adebiyi et al. [20], 1 g of each sample was weighed into a Teflon tube (MARSXpress – High Throughput Vessels, Matthews, USA) and mixed with 10 mL concentrated nitric acid (HNO_3_). A blank sample (without the sorghum samples) was also prepared from the HNO_3,_ and all samples were digested in a microwave digester (CEM One TouchTM Technology, CEM Technologies, Matthews, USA). The microwave-digester temperature conditions were as follows: temperature program was ramped to 180 °C for 10 min and kept at 180 °C for another 10 min, followed by immediate ventilation at room temperature for 20 min. The resulting solutions were cooled and made up to mark with Milli-Q water (Millipore, Bedford, USA) in a 50 mL volumetric flask.

#### 2.4.2 ICP-OES and ICP-MS

Stock and working standard solutions were prepared by using the National Institute of Standards and Technology (NIST) traceable certified reference materials (CRMs) of calcium (Ca), chromium (Cr), copper (Cu), iron (Fe), potassium (K), lead (Pb), magnesium (Mg), manganese (Mn), molybdenum (Mo), sodium (Na), phosphorus (P), sulphur (S) and zinc (Zn). Extracts were analyzed on ICP-OES equipment (Spectro ARCOS, Spectro Analytical Instruments, Kleve, Germany) under the instrumental conditions and parameters of radio frequency power = 1400 W; Nebulizer type = Concentric; Nebulizer flow = 1.0 L/min; Sample flow = 1.0 mL/min and Rinse time = 5 min. For OES the elements and their wavelengths were Ca (396.847), Fe (259.941), K (766.491), Mg (279.553), P (177.495), S (180.731), and Na (589.592). The ICP-MS used was the PerkinElmer Nexion 300X Q-ICP-MS (PerkinElmer (Pty), Midrand, South Africa). Radio frequency power = 1550 W; Nebulizer type = Concentric; Nebulizer flow = 0.8 L/min; Sample flow = 1.4 mL/min and Rinse time = 10 min. All elements were measured under KED mode with a helium flowrate of 5.2 mL/min. For MS, the elements Al (27), Mn (55), Co (59), Cr (52), Cu (63), Zn (66), Mo (98), and Pb (208) were used.

### 2.5 Functional properties

#### 2.5.1 Bulk density

The sorghum samples (50 g) were each weighed into a 100 mL measuring cylinder. The cylinder was tapped on a laboratory bench several times to a constant volume and then recorded. The bulk density (g/cm^3^) was expressed as the weight of the sample per volume of the sample after tapping [21].

#### 2.5.2 Water and oil absorption capacity (WAC and OAC)

One gram (1 g) of each respective sample was weighed in a centrifuge tube, and 10 mL distilled water or refined sunflower oil was added. This was mixed properly, kept at ambient temperature for 30 min (min) and then centrifuged (Eppendorf 5702R, Merck, Johannesburg, South Africa) at 2000×*g* for 10 min while the supernatant was decanted. The WAC and OAC were calculated as the difference between the initial weight of the sample and the weight of the sample after the water/oil added had been decanted. Results obtained were expressed on a dry basis as grams of water/oil bound per gram of flour sample [22].

#### 2.5.3 Swelling capacity

In 100 mL measuring cylinders, the respective samples were filled up to the 10 mL mark and distilled water was added to adjust the total volume to 50 mL. The top of the measuring cylinders were covered tightly and the solution was then mixed by inverting the cylinder. After 2 min, the suspension was inverted again and allowed to stand for a further 30 min while the volume occupied by the sample was taken after 30 min [23].

#### 2.5.4 Hydration capacity and hydration index

Using the methods described by Aditi and Arivuchudar [24], 2 g of each sample was weighed in a centrifuge tube and 10 mL of distilled water was added. This was then properly mixed on a vortex for 2 min and was allowed to stand for 10 min. The mixture was centrifuged immediately, and the supernatant was decanted carefully, while the sediment was weighed. The hydration capacity was taken as the ratio of the weight of the sediment to the dry sample weight [(weight of sediment − weight of tube)/dry weight of sample), while the hydration index was calculated as [24]:

(2) Hydration index (HI) = hydration capacity/dry weight of the sample.

### 2.6 Statistical analysis

All experiments were conducted in triplicate and an analysis of variance (ANOVA) was done using SPSS version 20 (IBM, Armonk, USA). Means were separated using Tukey’s test and differences at a 5% (p ≤ 0.05) level were considered statistically significant.

## 3 Results and discussion

### 3.1 Proximate compositions and total energy of the sorghum flours and fractions

The proximate compositions of the white and brown sorghum flours are shown in Table 1. The moisture content of the white sorghum flour (WSF), white dehulled sorghum flour (WDF) and white sorghum bran (WSB) ranged from 8.29 to 10.30%, with values for WSF significantly (p ≤ 0.05) higher than that of the WSB and WDF. The moisture content of the brown sorghum flour (BSF), brown dehulled sorghum flour (BDF) and brown sorghum bran (BSB) ranged from 8.31 to 10.82%, with the BDF significantly (p ≤ 0.05) higher than that of the BSB and BSF samples. The varying moisture content between the respective fractions could be attributed to the dehulling process, while differences between WSF and BSF could be ascribed to the innate water holding capacity of each variety (also observed in Section 3.4). The moisture contents of the grains and other fractions were around 10% or less, which could suggest a longer shelf life and keeping quality [25].

**Table 1:** Proximate compositions of sorghum flours.

Parameters (%)	WSF	WDF	WSB	BSF	BDF	BSB
Moisture	10.30 ± 0.03^c^	10.16 ± 0.02^b^	8.29 ± 0.01^a^	10.26 ± 0.01^c^	10.82 ± 0.02^d^	8.31 ± 0.02^a^
Crude Protein	9.10 ± 0.05^b^	8.58 ± 0.01^a^	11.58 ± 0.01^e^	11.21 ± 0.01^d^	10.31 ± 0.02^c^	13.71 ± 0.01^f^
Crude Fat	2.42 ± 0.02^c^	1.28 ± 0.01^a^	5.34 ± 0.02^e^	3.15 ± 0.01^d^	1.74 ± 0.01^b^	5.71 ± 0.01^f^
Crude Fiber	2.18 ± 0.02^c^	0.57 ± 0.02^a^	8.25 ± 0.01^e^	2.00 ± 0.01^b^	0.55 ± 0.02^a^	6.06 ± 0.02^d^
Ash	1.20 ± 0.01^c^	0.59 ± 0.01^a^	2.36 ± 0.02^e^	1.17 ± 0.01^c^	0.70 ± 0.01^b^	2.23 ± 0.02^d^
Carbohydrate	74.8 ± 0.03^c^	78.82 ± 0.01^d^	64.18 ± 0.02^a^	72.21 ± 0.01^b^	75.88 ± 0.01^c^	63.98 ± 0.01^a^
Energy (kcal)	357.38 ± 0.03^b^	361.12 ± 0.01^c^	351.10 ± 0.02^a^	362.03 ± 0.01^d^	360.42 ± 0.01^c^	362.15 ± 0.02^d^

WSF - white whole grain sorghum; WDF - white dehulled sorghum; WSB - white sorghum bran; BSF - brown whole grain sorghum; BDF - brown dehulled sorghum; BSB - brown sorghum bran. *Each value is a mean of triplicates ± SD of triplicates. Means with no common letters within a row significantly differ (p ≤ 0.05).

The crude protein content of the brans (WSB and BSB) was 11.58% and 13.71%, respectively, which was higher than those of the other fractions. The dehulled flours had the least protein content with 8.58% and 10.31% for WDF and BDF, respectively. The removal of the pericarp (which contains most of the fibre and minerals) in the dehulled samples might have contributed to the overall reduction of protein, while the protein differences between the dehulled samples (WDF and BDF) could be ascribed to the types, genetics, and growing conditions of white and brown sorghum varieties [26,27]. The protein content of Asian and African sorghum cultivars has been reported to be 10.02−14.00% respectively [28] and protein contents in yellow, white and four hybrid sorghum samples were found to be between 6.23 and 13.81% [29]. The levels of proteins reported in these studies concurs with the protein content found in this study (Table 1). Dehulling mostly removes the pericarp, some of the germ and part of the outer endosperm, which are known to contain protein, and this therefore reduces the amount of protein available in the grain [30]. Accordingly, the use of whole grains is preferable to refined grains in developing gluten-free products, as the whole grain contributes to a better, more beneficial product [31].

The crude fat content of the samples ranged between 1.28 and 5.71% and was significantly (p ≤ 0.05) higher in the bran samples (Table 1) while the decrease in the dehulled samples might be due to the removal of fat-rich germ along with the bran [27]. Sorghum fats are situated in the scutellum, and can reduce significantly when kernels are degermed or dehulled [32]. Fat plays a key role in the shelf life of food products and could be undesirable as it can promote rancidity in foods, leading to the development of odorous and unpleasant compounds [33,34]. However, the fat in cereal grains is linked to the amount of energy the grain contains, and, as a result, whole grain cereals are preferable due to their high content of unsaturated fats, which help prevent obesity, diabetes, and cardiovascular diseases [35].

The ash contents of the WDF and BDF samples were significantly (p ≤ 0.05) lower than those of the other samples (WSF, WSB, BSF and BSB). The ash content indicates the total amount of minerals present within a food. The low ash content in the dehulled sorghum samples might be attributed to the removal of the hull or seedcoat from the endosperm. Sorghum hulls are characterized by their high crude protein, crude fibre, and ash content [36]. Ash is mainly found in the bran, which is removed during the dehulling process. Ash can be used as an indicator of bran contamination in cereal flour production, particularly in wheat [37,38]. Espinosa-Ramírez et al. [39] reported that dehulled sorghum flour contained less ash, fat, and protein than whole grains (Table 1 shows the similar findings in this study). They attributed this to the removal of the pericarp, aleurone and germ tissues. The bran samples in this study were high in ash content and had higher mineral contents (Section 3.3).

The lower crude fibre content of the dehulled sorghum samples could be linked to the removal of the pericarp, which contains high crude fibre [40]. As with the ash content, the removal of the pericarp seems to have resulted in the removal of fibre-rich constituents, which as observed in Table 1 was much higher in the bran (WSB and BSB). Low fibre products are beneficial in weaning foods, as high fibre in these foods cause gut mucosa irritation, as well as lesser nutrient availability and digestibility. However, fractions with higher fibre may be desirable in adult nutrition. The presence of high fibre in the bran samples (Table 1) could benefit diabetic patients, as it has been reported that high fibre improves glucose tolerance [41]. The carbohydrate content of WSF, WDF and WSB ranged from 64.18 to 78.82%, while for BSF, BDF and BSB, the carbohydrate content ranged from 63.98 to 75.88%. The values obtained for the whole grain are also similar to those reported for whole sorghum grains cultivar BRS 310 [31]. As with the other constituents investigated, the carbohydrate values of the various anatomical parts were different, with the dehulled samples having significantly higher levels (WDF with 78.82 and BDF with 75.88). Such high levels in the dehulled samples could be ascribed to higher levels of starch and sugars concentrated in the embryo of the grains, which became more accessible due to the dehulling process. The energy values of the brown sorghum samples were generally higher than those of the white sorghum samples, which could be ascribed to varietal differences.

### 3.2 Amino acid (AA) compositions of sorghum flours

The amino acid (AA) compositions of the whole grain, dehulled and bran sorghum flours are presented in Table 2. The AAs of both sorghum varieties were generally similar except for leucine, phenylalanine, valine, glutamic acid and proline. Such similarities could be attributed to the commonality of the grains, while differences could be ascribed to genetic and environmental factors, as well as agronomic conditions during plant growth [42]. The bran samples however, were, observed to have significantly (p ≤ 0.05) higher levels of essential and non-essential AAs. Glutamic acid, proline, alanine, leucine, aspartic acid, glycine, and arginine were the most dominant AAs of the sorghum brans (Table 2). Leucine, phenylalanine and valine, which were significantly higher in the brown variety, are essential AAs. This is an indication that the BSB could be good sources of these AAs, as they cannot be synthesized by the body and are only obtained from food sources. It is to be noted that carotenoids and anthocyanins are the major groups of pigment-related compounds conferring colouration to grain varieties [43,44], and it could be postulated that these compounds could have positively contributed to the AA levels in the brown varieties. This author further explained the correlation between amino acids and antioxidants (anthocyanins) in grains [45]. It was noted that higher retention of amino acids in coloured wheat may be caused by anthocyanins' ability to cover up and shield proteins and amino acids from oxidative and thermal degradation [45]. As this factor was not investigated in this study, additional study is needed to further ascertain the correlation between the grain colour and recorded levels of these AAs.

**Table 2:** Amino acid compositions of sorghum flours.

Parameters (g/100 g)	WSF	WDF	WSB	BSF	BDF	BSB
**Essential**						
Histidine	0.45 ± 0.02^c^	0.26 ± 0.01^a^	0.54 ± 0.02^d^	0.42 ± 0.02^b^	0.40 ± 0.02^b^	0.46 ± 0.02^c^
Isoleucine	0.46 ± 0.02^b^	0.41 ± 0.02^a^	0.55 ± 0.02^d^	0.52 ± 0.02^d^	0.49 ± 0.02^c^	0.65 ± 0.02^e^
Leucine	0.39 ± 0.02^a^	1.08 ± 0.04^b^	1.20 ± 0.04^c^	1.39 ± 0.05^e^	1.32 ± 0.02^d^	1.60 ± 0.04^f^
Lysine	0.30 ± 0.01^c^	0.17 ± 0.03^a^	0.44 ± 0.02^e^	0.24 ± 0.03^b^	0.26 ± 0.03^b^	0.41 ± 0.02^d^
Methionine	0.09 ± 0.01^b^	0.06 ± 0.02^a^	0.09 ± 0.01^b^	0.10 ± 0.01^b^	0.10 ± 0.01^b^	0.12 ± 0.01^c^
Phenylalanine	0.48 ± 0.02^b^	0.44 ± 0.02^a^	0.54 ± 0.02^c^	0.57 ± 0.03^d^	0.53 ± 0.03^c^	0.69 ± 0.02^e^
Threonine	0.34 ± 0.01^b^	0.28 ± 0.02^a^	0.41 ± 0.02^d^	0.39 ± 0.02^c^	0.34 ± 0.03^b^	0.51 ± 0.03^e^
Valine	0.55 ± 0.02^b^	0.47 ± 0.02^a^	0.69 ± 0.02^d^	0.63 ± 0.03^c^	0.56 ± 0.03^b^	0.80 ± 0.02^e^
**Non-essential**						
Alanine	0.80 ± 0.02^b^	0.73 ± 0.05^a^	0.91 ± 0.02^c^	1.00 ± 0.02^d^	0.89 ± 0.02^c^	1.17 ± 0.03^e^
Arginine	0.47 ± 0.02^d^	0.33 ± 0.03^a^	0.66 ± 0.03^e^	0.44 ± 0.02^c^	0.38 ± 0.02^b^	0.68 ± 0.03^e^
Aspartic acid	0.72 ± 0.03^c^	0.57 ± 0.04^a^	0.92 ± 0.06^e^	0.76 ± 0.03^d^	0.67 ± 0.02^b^	1.01 ± 0.03^f^
Glutamic acid	1.97 ± 0.03^b^	1.80 ± 0.03^a^	2.14 ± 0.03^c^	2.46 ± 0.03^e^	2.23 ± 0.03^d^	2.76 ± 0.04^f^
Glycine	0.35 ± 0.02^c^	0.24 ± 0.03^a^	0.51 ± 0.03^d^	0.34 ± 0.03^c^	0.29 ± 0.01^b^	0.51 ± 0.03^d^
Proline	0.75 ± 0.03^b^	0.67 ± 0.02^a^	0.81 ± 0.03^c^	0.89 ± 0.02^d^	0.82 ± 0.03^c^	0.99 ± 0.03^e^
HO-Proline	0.01 ± 0^a^	0.01 ± 0^a^	0.04 ± 0^b^	0.01 ± 0^a^	0.01 ± 0^a^	0.02 ± 0^a^
Serine	0.45 ± 0.02^b^	0.37 ± 0.02^a^	0.57 ± 0.03^d^	0.53 ± 0.03^c^	0.47 ± 0.02^b^	0.67 ± 0.02e
Tyrosine	0.42 ± 0.02^c^	0.37 ± 0.02^a^	0.46 ± 0.02^d^	0.39 ± 0.02^b^	0.45 ± 0.01^d^	0.52 ± 0.02^e^

g/100 g – gram/100 g. WSF - white whole grain sorghum; WDF - white dehulled sorghum; WSB - white sorghum bran; BSF - brown whole grain sorghum; BDF - brown dehulled sorghum; BSB - brown sorghum bran. Means with no common letters within a row significantly differ (p ≤ 0.05).

The generally higher levels of AA content identified in the bran samples are related to the high protein content of sorghum bran (Table 1). This suggests that most of the proteins, and consequently amino acids, are more concentrated in the pericarp of the grains, irrespective of the variety. During dehulling of sorghum, the germ and pericarp are usually removed. However, these components are two to three times richer in lysine than the endosperm [[46], [47], [48]]. The effect of such removal is observed in the reduced lysine content and certain other AAs in the dehulled samples (Table 2). Although the AA levels in BSF were generally higher than those of the WSF, histidine, lysine, arginine and tyrosine were significantly higher in WSF. Interestingly, three of these amino acids (histidine, lysine and arginine) have basic side chains at neutral pH and this uniqueness in their structure could have contributed to their higher levels in WSF. It should, however, be noted that cereal grains are generally poor sources of lysine and must be supplemented with legumes (or other protein sources) which are rich in proteins and amino acids. The results of this study indicate that the whole grains and different anatomical parts (dehulled and bran) of sorghum can be used to prepare gluten-free products, especially in combination with other high-protein sources, to address protein energy malnutrition [49]. As further opined by Cayres et al. [50], incorporating such raw materials in food preparations will contribute to dietary diversity and sustainability.

### 3.3 Mineral compositions of sorghum flours

The absorption of mineral elements in cereals differs depending on the environmental and genotypic impacts, and the degree of technological processing [51]. The mineral compositions of WSF, WDF, WSB, BSF, BDF and BSB are presented in Table 3. Differences in their levels can be attributed to genotypic differences and the impact of dehulling, resulting in different anatomical fractions. Particularly with regard to the variances between WSF and BSF, differences in agronomic conditions as well as nutrient composition of the soil and how these nutrients were bioaccumulated by the respective plants during grain formation would have influenced these values. The results in Table 1 show that the bran samples contained higher amounts of ash than the inner parts of the kernels, and this is further reflected in Table 3, with the bran generally containing higher levels of minerals. This inevitably gives credence to the general supposition that most minerals are accumulated on the outer part of a grain and, as observed in this study, the process of dehulling reduces the levels of minerals, irrespective of the grain type. However, although not investigated in this study, a clear advantage of dehulling as reported in available literature is the reduction in antinutrient levels [52,53].

**Table 3:** *Mineral compositions* (mg/100 g) *of sorghum flours*.

Minerals	WSF	WDF	WSB	BSF	BDF	BSB
Al	0.03 ± 0^b^	0.02 ± 0^a^	0.10 ± 0^c^	0.04 ± 0^b^	0.01 ± 0^a^	0.11 ± 0^c^
Ca	463.21 ± 16.8^c^	358.35 ± 8.21^b^	995.17 ± 38.72^e^	474.91 ± 26.87^d^	253.15 ± 5.39^a^	1020.91 ± 21.48^f^
Co	0.01 ± 0^a^	0.01 ± 0^a^	0.02 ± 0^a^	0.02 ± 0^a^	0.01 ± 0^a^	0.02 ± 0^a^
Cr	0.02 ± 0^a^	0.02 ± 0^a^	0.09 ± 0^c^	0.05 ± 0^b^	0.01 ± 0^a^	0.18 ± 0.01^d^
Cu	1.31 ± 0.01^c^	1.27 ± 0.03^b^	3.09 ± 0.03^d^	1.25 ± 0.02^b^	0.87 ± 0.11^a^	3.07 ± 0.06^d^
Fe	0.26 ± 0.01^b^	0.26 ± 0.01^b^	0.42 ± 0.01^d^	0.32 ± 0.01^c^	0.24 ± 0.01^a^	0.50 ± 0.01^e^
K	2.29 ± 0.02^c^	2.36 ± 0.01^d^	3.65 ± 0.04^f^	2.25 ± 0.01^b^	1.92 ± 0.03^a^	3.48 ± 0.06^e^
Mg	104.96 ± 3.40^b^	134.45 ± 3.37^d^	226.02 ± 5.45^e^	130.91 ± 2.24^c^	92.72 ± 1.94^a^	292.25 ± 7.03^f^
Mn	0.98 ± 0.01^b^	1.02 ± 0.01^c^	2.30 ± 0.03^e^	1.10 ± 0.02^d^	0.69 ± 0.01^a^	2.61 ± 0.03^f^
Mo	0.09 ± 0^b^	0.09 ± 0^b^	0.17 ± 0^d^	0.05 ± 0^a^	0.04 ± 0^a^	0.10 ± 0^c^
Na	4.84 ± 0.07^c^	4.77 ± 0.10^b^	4.74 ± 0.07^b^	4.81 ± 0.08^c^	4.74 ± 0.11^b^	4.69 ± 0.07^a^
P	0.37 ± 0.01^d^	0.27 ± 0.01^b^	0.57 ± 0.02^e^	0.30 ± 0^c^	0.22 ± 0^a^	0.66 ± 0.01^f^
Pb	0.19 ± 0.01^a^	0.17 ± 0.01^a^	0.32 ± 0.01^c^	0.18 ± 0^a^	0.17 ± 0^a^	0.27 ± 0^b^
S	0.20 ± 0.01^b^	0.18 ± 0.01^a^	0.29 ± 0^e^	0.25 ± 0.01^d^	0.21 ± 0.01^c^	0.33 ± 0^f^
Zn	0.46 ± 0^b^	0.51 ± 0.01^c^	1.03 ± 0.01^e^	0.63 ± 0^d^	0.37 ± 0.01^a^	1.41 ± 0.02^f^

mg/100 g – milligram/100 g; WSF - white whole grain sorghum; WDF - white dehulled sorghum; WSB - white sorghum bran; BSF -brown whole grain sorghum; BDF - brown dehulled sorghum; BSB - brown sorghum bran. Al – aluminum; Ca – calcium; Cr – chromium; Cu – copper; Fe – iron; K – potassium; Mg – magnesium; Mn – manganese; Mo – molybdenum; Na – sodium; P – phosphorus; S – sulphur; Zn – zinc. Each value is a mean of triplicates ± SD of triplicates. Means with no common letters within a row significantly differ (p < 0.05).

It has already been established that brans are rich sources of minerals such as iron (Fe), magnesium (Mg), manganese (Mn), phosphorus (K), and zinc (Zn) [54], which concurs with the findings in this study. The Fe content was observed to reduce in the BDF, although there was no significant difference (p ≤ 0.05) observed between WSF and WDF (Table 3). This might be attributed to the fact that iron in the sorghum kernel is very well absorbed in the outer layers of the kernel [13], while the same mechanism has also been reported for pearl millet [55]. It has been reported that there is about 45% and 50% loss of iron in sorghum and pearl millet, respectively, at a 10% dehulling rate [56]. Likewise, a 96% loss of iron reduction was observed across 12 fonio (*Digitaria exilis*) landraces which were dehulled followed by sieving and washing [57]. The reduction of the minerals in dehulled grains can also be attributed to the absorption of mineral substances primarily in the upper layers of the grain, which are then separated in the process of peeling the grains [51].

Zinc was found to decrease in the BDF, and an increase in zinc was observed in the WDF compared to WSF (Table 3). Such an increase could possibly be ascribed to redistribution during dehulling and the fact that a lesser amount possibly migrated into the cotyledon. In contrast, there has been a reported loss of about 25% and 5% zinc in sorghum and pearl millet, respectively at a 10% dehulling rate [55,56]. Contamination from equipment while milling these grains can also greatly affect the mineral content of the resulting flour [13]. Calcium was found in a high amount in the WSB (995.17 mg/100 g) and BSB (1020.91 mg/100 g) samples. These were also recorded for the WDF (995.17 mg/100 g) and BDF (1020.91 mg/100 g) as well as WSF (995.17 mg/100 g) and BSF (1020.91 mg/100 g) samples (Table 3). Selenium and cadmium were not detected in this study. Cadmium is a lethal non-vital heavy metal that does not play a role in the biological process of living organisms, even in small concentrations [58].

### 3.4 Functional properties of sorghum flours

The bulk density reported in this study (Table 4) showed that the white and brown dehulled sorghum flours had significantly (p ≤ 0.05) higher values compared to the other sorghum flours. The lowest values (0.50 and 0.58 g/cm^3^) of bulk density were recorded for the white and brown sorghum brans respectively (Table 4). This could be due to the absence of complex structures such as carbohydrates and proteins in the bran. The dehulled samples, however, contained these components in the germ, as reflected in their higher bulk densities. The higher bulk densities of the dehulled samples could also be related to the carbohydrate contents, which were higher in the dehulled samples (Table 1). They could also be linked to the moisture content of the samples [59]. A study on finger millet reported a slightly higher bulk density in decorticated millet over native millet, and this was most likely caused by the removal of the seed coat and by the kernel’s reduced porosity [60]. Another factor that may have contributed to a rise in bulk density is the elimination of the relatively lighter bran layer, which is rich in lipids and has a lower specific gravity than endosperm components such as starch and proteins [61]. While flours of high densities suggest suitability for food preparations [59], those of lower bulk density would be useful in formulating complementary foods [62].

**Table 4:** Functional properties of sorghum flours.

Parameters	WSF	WDF	WSB	BSF	BDF	BSB
Bulk density (g/cm^3^)	0.88 ± 0.01^d^	0.97 ± 0.01^f^	0.50 ± 0.01^a^	0.83 ± 0.01^c^	0.92 ± 0.01^e^	0.58 ± 0.01^b^
OAC (g/g)	15.38 ± 0.14^a^	15.33 ± 0.21a	16.09 ± 0.13^c^	15.42 ± 0.03^b^	15.15 ± 0.07^a^	15.44 ± 0.11^b^
WAC (g/g)	15.51 ± 0.24^a^	15.44 ± 0.21^a^	16.97 ± 0.21^b^	15.46 ± 0.08^a^	15.30 ± 0.20^a^	16.35 ± 0.24^b^
Swelling capacity (g/ml)	1.02 ± 0.01^a^	1.02 ± 0.01^a^	1.07 ± 0.01^b^	1.02 ± 0.01^a^	1.04 ± 0.01^a^	1.02 ± 0.01^a^
Hydration capacity (g/ml)	2.19 ± 0.02^a^	2.03 ± 0.03^a^	2.95 ± 0.05^b^	2.31 ± 0.14^a^	2.17 ± 0.04^a^	2.76 ± 0.10^a^
Hydration index (%)	1.09 ± 0.01^a^	1.02 ± 0.02^a^	1.47 ± 0.02^d^	1.15 ± 0.07^b^	1.09 ± 0.02^a^	1.38 ± 0.05^c^

*Each value is a mean of triplicates ± SEM of triplicates. Means with no common letters within a row significantly differ (p < 0.05). OAC – oil absorption capacity; WAC – water absorption capacity.

Oil and water absorption capacity, hydration capacity and hydration index were higher in the bran samples than in the other sorghum flours (Table 4). Water absorption capacity (WAC) is a representation of the ability of a food product to blend or merge with water in instances where water is limited. Low and high values of WAC could be attributed to structure compactness and loss of structure of the starch polymers respectively [63,64]. Another study reported that lower WAC might be linked to the lower availability of polar amino acids in the flours [65], and this was the case in this study, as glutamic acid, threonine, serine, and histidine levels (polar AAs) were lower in the whole grain and dehulled sorghum flours (Table 2). The low WAC in the dehulled samples may also be attributed to the property of fibre to bind and hold water [66]. It is worth noting that hydrophilic polysaccharides may be lost after the husk is removed, lowering the WAC of flours made from polished grains [67]. The oil absorption capacity (OAC) is vital during food preparation, to enhance flavour and mouth feel. A possible reason for the high levels of OAC in the bran samples might be due to a higher fat content in the sorghum bran flours (Table 1). Differences in the OAC and WAC could also be reflected in the proximate composition described in Section 3.1. The varietal differences observed in this study may have contributed to the differences in the starch composition, carbohydrate, and protein related structures as well as the fat-related components, all of which contribute to the functional properties of the grains and their respective fractions. As indicated by Suresh and Samsher [68], the swelling capacity of flours depends on the processing techniques as well as the variety, among other factors. Although not significantly (p ≤ 0.05) different, the swelling capacity of the samples in this study varied slightly from each other. These slight, non-significant difference could thus be related to varietal differences and possible similarities in the swelling-related constituents of the samples.

## 4 Limitations

A number of limitations in this study which exceeded the current scope could be further explored in future studies. These include (i) many more varieties of sorghum need to be evaluated to further understand the influences of dehulling and composition of these different anatomical parts, (ii) different dehulling rates should be tested, and the moisture contents of the grain could possibly be varied before dehulling to investigate the effects of these on the composition and properties of the different anatomical parts, (iii) other constituents including pigment related components (carotenoids, anthocyanins), phenolic compounds and health promoting properties, and antinutritional factors should be investigated and (iv) use of these different anatomical parts in food product development should be explored.

## 5 Conclusion

An investigation of the nutritional and functional properties of two sorghum types and their anatomical parts was provided in this study. The composition of the two sorghum types in this study are relatively similar, although some properties proved to be different. It was nevertheless observed that both the white and brown sorghum bran flours contained significant amounts of nutrients and functional properties compared to the other seed fractions. While species similarity could explain the closeness of the values for the whole grains, the colour in some instances seems to have contributed to the differences in whole grain composition. On the other hand, dehulling was observed to significantly contribute to differences recorded for the different anatomical parts. Although the bran seems to have some better constituents, their potential antinutrients need to be investigated and possibly reduced to ensure optimum use in food product development. Further studies varying different dehulling rates should be explored to develop further insights into the influence of dehulling levels on composition. Equally important is research looking at the development of food products with sorghum bran, and subsequent characterization of the overall composition and acceptability of such products. Finally, investigation into the health-promoting constituents of these food fractions should be done, as further insights will provide much-needed information on different anatomical sorghum parts.

## Author contribution statement

**Janet Adeyinka Adebo:** Conceived and designed the experiments; Analyzed and interpreted the data; Contributed reagents, materials, analysis tools or data; Wrote the paper.

**Hema Kesa:** Analyzed and interpreted the data; Contributed reagents, materials, analysis tools or data.

## Data availability statement

Data will be made available on request.

## Declaration of competing interest

The authors declare that they have no known competing financial interests or personal relationships that could have appeared to influence the work reported in this paper
